# A household-level score to predict the risk of tuberculosis among contacts of patients with tuberculosis: a derivation and external validation prospective cohort study

**DOI:** 10.1016/S1473-3099(19)30423-2

**Published:** 2020-01

**Authors:** Matthew J Saunders, Tom Wingfield, Sumona Datta, Rosario Montoya, Eric Ramos, Matthew R Baldwin, Marco A Tovar, Benjamin E W Evans, Robert H Gilman, Carlton A Evans

**Affiliations:** a Department of Infectious Diseases, Imperial College London, London, UK; b Innovation For Health And Development, Laboratory of Research and Development, Universidad Peruana Cayetano Heredia, Lima, Peru; c Innovación Por la Salud Y Desarrollo, Asociación Benéfica PRISMA, Lima, Peru; d Department of Clinical Infection, Microbiology and Immunology, Institute of Infection and Global Health, University of Liverpool, UK; e LIV-TB Collaboration and Department of Clinical Science, Liverpool School of Tropical Medicine, Liverpool, UK; f Social Medicine, Infectious Diseases and Migration Group, Department of Public Health Sciences, Karolinska Institute, Stockholm, Sweden; g College of Physicians and Surgeons, Columbia University, New York, NY, USA; h Johns Hopkins Bloomberg School of Public Health, Baltimore, MD, USA

## Abstract

**Background:**

The epidemiological impact and cost-effectiveness of social protection and biomedical interventions for tuberculosis-affected households might be improved by risk stratification. We therefore derived and externally validated a household-level risk score to predict tuberculosis among contacts of patients with tuberculosis.

**Methods:**

In this prospective cohort study, we recruited tuberculosis-affected households from 15 desert shanty towns in Ventanilla and 17 urban communities in Callao, Lima, Peru. Tuberculosis-affected households included index patients with a new diagnosis of tuberculosis and their contacts who reported being in the same house as the index patient for more than 6 h per week in the 2 weeks preceding index patient diagnosis. Tuberculosis-affected households were not included if the index patient had no eligible contacts or lived alone. We followed contacts until 2018 and defined household tuberculosis, the primary outcome, as any contact having any form of tuberculosis within 3 years. We used logistic regression to identify characteristics of index patients, contacts, and households that were predictive of household tuberculosis, and used these to derive and externally validate a household-level score.

**Findings:**

Between Dec 12, 2007, and Dec 31, 2015, 16 505 contacts from 3 301 households in Ventanilla were included in a derivation cohort. During the 3-year follow-up, tuberculosis occurred in contacts of index patients in 430 (13%, 95% CI 12–14) households. Index patient predictors were pulmonary tuberculosis and sputum smear grade, age, and the maximum number of hours any contact had spent with the index patient while they had any cough. Household predictors were drug use, schooling of the female head of a household, and lower food spending. Contact predictors were if any of the contacts were children, number of lower-weight (body-mass index [BMI] <20·0 kg/m^2^) adult contacts, number of normal-weight (BMI 20·0–24·9 kg/m^2^) adult contacts, and number of past or present household members who previously had tuberculosis. In this derivation cohort, the score *c* statistic was 0·77 and the risk of household tuberculosis in the highest scoring quintile was 31% (95% CI 25–38; 65 of 211) versus 2% (95% CI 0–4; four of 231) in the lowest scoring quintile. We externally validated the risk score in a cohort of 4248 contacts from 924 households in Callao recruited between April 23, 2014, and Dec 31, 2015. During follow-up, tuberculosis occurred in contacts of index patients in 120 (13%, 95% CI 11–15) households. The score *c* statistic in this cohort was 0·75 and the risk of household tuberculosis in the highest scoring quintile was 28% (95% CI 21–36; 43 of 154) versus 1% (95% CI 0–5; two of 148) in the lowest scoring quintile. The highest-scoring third of households captured around 70% of all tuberculosis among contacts. A simplified risk score including only five variables performed similarly, with only a small reduction in performance.

**Interpretation:**

This externally validated score will enable comprehensive biosocial, household-level interventions to be targeted to tuberculosis-affected households that are most likely to benefit.

**Funding:**

Wellcome Trust, Medical Research Council, Department of Health and Social Care, Department for International Development, Joint Global Health Trials consortium, Bill & Melinda Gates Foundation, Innovation for Health and Development.

## Introduction

Tuberculosis-affected households have intense tuberculosis transmission, with contacts of index patients with tuberculosis at substantially higher risk of latent tuberculosis infection and tuberculosis disease than other community members.^1^ Although the majority of tuberculosis transmission might occur outside the household, comprehensive interventions targeted to tuberculosis-affected households have the potential to reduce the population-level burden of tuberculosis.^2^, ^3^, ^4^ Such interventions include tuberculosis screening, surveillance, and preventive treatment for contacts, integrated with social protection interventions to incentivise and enable access to health care and concurrently reduce poverty-related tuberculosis risk factors.^5^, ^6^, ^7^

**Research in context**

**Evidence before this study**

We searched PubMed, Google Scholar, Web of Science, and Embase databases for studies published between database inception and Jan 1, 2019, that characterised the epidemiology, risk factors, and management of contacts of patients with tuberculosis. Although the majority of tuberculosis transmission probably occurs outside of households, contacts who live with patients with tuberculosis are a readily accessible group known to be at high risk of latent tuberculosis infection and tuberculosis disease. Consequently, social and biomedical interventions targeted to contacts in tuberculosis-affected households might have a population-level effect on tuberculosis burden. Despite strong evidence of their value, these interventions are infrequently implemented in many settings because of a lack of programmatic focus on tuberculosis prevention and limited resources. To help address this challenge, we previously derived and externally validated a risk score that predicts the risk of tuberculosis among individual adult contacts of patients with laboratory confirmed tuberculosis, independently of baseline latent tuberculosis infection status. However, because tuberculosis is a disease that inherently clusters in households, commonly affecting multiple household members, a score that predicts household tuberculosis (tuberculosis occurring in any contact within the household) might have more pragmatic value to public health programmes through identifying households most likely to benefit from comprehensive biosocial, household-level interventions. To our knowledge, no such score exists.

**Added value of this study**

In this study, we derived and externally validated a score that predicted and stratified tuberculosis-affected households with large differences in the risk of household tuberculosis. This score combines data on easily recordable index patient, household, and contact characteristics into a model that can be used at the time of index patient diagnosis to prioritise enhanced active case finding for contacts, preventive treatment, and social protection interventions. Prioritisation using this score should maximise the impact of active case finding and preventive treatment and concurrently address poverty-related risk factors that drive the tuberculosis epidemic and cluster in tuberculosis-affected households. Although these interventions should be considered for all tuberculosis-affected households, their epidemiological effect and cost-effectiveness is likely to be improved by risk stratification, especially in resource-constrained settings.

Our score shows that relatively wealthier households, in which the index patient has extra-pulmonary tuberculosis and the majority of contacts are normal weight or overweight adults, have a risk of household tuberculosis of about one in 100. In contrast, poorer, previously tuberculosis-affected households, in which the index patient has pulmonary tuberculosis with a high sputum smear grade and the majority of contacts are children or lower weight adults, have a risk of household tuberculosis of about one in four. In different settings our score can be used in diverse ways. For example, prioritising the highest scoring third of households would capture more than 70% of the overall tuberculosis burden among contacts, and prioritising the highest scoring two-thirds of households would capture more than 90% of the overall tuberculosis burden among contacts. This score is freely available to use and can be found on the Innovation For Health And Development website.

**Implications of all the available evidence**

Tuberculosis elimination will only be possible with the scale-up of tuberculosis case-finding and prevention and the integration of biomedical interventions with social protection interventions to address the underlying determinants driving the tuberculosis epidemic. Our risk score represents a step forward for tuberculosis care and prevention, enabling public health programmes to prioritise comprehensive biosocial, household-level interventions for tuberculosis-affected households at high risk of tuberculosis among contacts. Future research should focus on further external validation, refinement, and impact evaluation of this score in settings with different epidemiology, health behaviours, and household characteristics.

The implementation of these interventions in tuberculosis-affected households has been hampered by a lack of focus on prevention by national tuberculosis programmes, principally because resources have historically been prioritised for tuberculosis diagnosis and treatment.^8^, ^9^ Although this strategy has helped save millions of lives, the impact on tuberculosis incidence has not yet been detectable. Furthermore, tuberculosis elimination is widely recognised as only being possible with the scale up of tuberculosis prevention and the integration of biomedical interventions with social protection interventions to address the underlying social determinants driving the tuberculosis epidemic.^10^

In previous work,^11^ we derived and externally validated a score to predict individual risk of tuberculosis among adult contacts of patients with infectious tuberculosis. This individual score, which includes clinical and sociodemographic risk factors, predicts tuberculosis independently of baseline latent tuberculosis infection status and is being evaluated within the ongoing Community Randomised Evaluation of a Socioeconomic Intervention to Prevent Tuberculosis trial to facilitate targeted tuberculosis screening and preventive treatment.^12^, ^13^, ^14^ However, our experiences during that trial suggest that a score might be of greater pragmatic value to public health programmes if, rather than identifying individual contacts at high risk of tuberculosis, it identified households in which contacts are at high risk of tuberculosis that could then be prioritised for comprehensive, household-level interventions.^15^ Furthermore, because tuberculosis is a disease that clusters in households, a household-level risk score might be a more effective method of prioritising resources to maximise epidemiological impact. In this much larger study, we used data on index patient, household, and contact characteristics to derive and externally validate such a score. As in our previous work, we aimed to derive a score that could be used without testing for latent tuberculosis infection because these tests are poor predictors of incident tuberculosis among contacts and are hampered by logistical and technical barriers to implementation.^8^, ^16^

## Methods

### Study design and participants

In this prospective cohort study, we collected data from two cohorts of tuberculosis-affected households from northern Lima, Peru, to derive and externally validate a household-level risk score for predicting tuberculosis occurrence in any contact. Households were recruited from the 15 desert shanty towns comprising Ventanilla district between Dec 12, 2007, and Dec 31, 2015, for the derivation cohort, and from 17 urban communities in Callao between April 23, 2014, and Dec 31, 2015, for the external validation cohort. Callao and Ventanilla are distinct geographical areas and have marked differences in population demographics and material living conditions, which are described in detail in our previous work.^11^

For both cohorts, tuberculosis-affected households included index patients with tuberculosis who were registered to receive treatment in health centres run by the Peruvian Ministry of Health and their contacts who reported being in the same house as the index patient for more than 6 h per week in the 2 weeks preceding index patient diagnosis. Contacts were not eligible if they were already taking tuberculosis treatment at the time the index patient started treatment or they were known to have received at least 4 weeks of isoniazid preventive treatment because of current exposure to the index patient. All contacts were offered free tuberculosis screening at the health centres (appendix pp 1–3). Households were not included if the index patient had no eligible contacts or lived alone.

All index patients gave written, informed consent and, when possible in the case of minors, assent to participate on behalf of their household. Ethical approval for the study was obtained from the Callao Ministry of Health, Asociación Benéfica PRISMA, and Imperial College London.

### Procedures

Study nurses worked in collaboration with local health centres to recruit index patients as soon as they were diagnosed with tuberculosis. Index patients were invited to give a sputum sample, which was tested by a combination of smear microscopy, an in-house microscopic-observation drug-susceptibility assay,^17^ an in-house MDR/XDR-TB Colour Test thin-layer agar assay,^18^ and the GeneXpert MTB/RIF assay (Cepheid, Maurens-Scopont, France). Study nurses then interviewed the index patient in the health centre to complete a demographic census of all eligible contacts and a questionnaire to obtain baseline characteristics of the index patient, household, and contacts.

### Outcomes

The primary outcome was household tuberculosis, a binary outcome defined as positive if any of the contacts in a household were known to have started tuberculosis treatment or been diagnosed with pulmonary or extra-pulmonary tuberculosis within 3 years of the date that the index patient started treatment. We censored follow up at 3 years because our previous work has shown that the majority of tuberculosis among contacts occurs within that timeframe.^20^ Household tuberculosis was ascertained by cross-referencing our contact census with tuberculosis treatment registers at health centres in the study setting using national identifier numbers and names. Identifier numbers were available for about 50% of contacts and, if names were used, they were cross referenced with age or date of birth to ensure accuracy. For households known to have had household tuberculosis, time to tuberculosis was defined from the date the index patient initiated treatment until the first date a contact in a household started treatment for, or was diagnosed with, tuberculosis.

### Statistical analysis

Analyses were based on all available data from an ongoing cohort study, so power calculations were not done. Continuous data were plotted and summarised by their medians and IQRs, because their distributions were non-parametric. Categorical data were summarised as proportions. We compared baseline characteristics between the derivation and external validation cohorts using the Mann-Whitney *U* test for continuous data and the χ^2^ test for categorical data. All analyses were done using Stata version 13, and all p values were two-sided and considered significant if less than 0·05.

We investigated the association between index patient, household, and contact variables and household tuberculosis in univariable logistic regression models, including data from households recruited to the derivation cohort. Details on how potential predictor variables were defined, transformed, and modelled (including multiple imputation methods to replace missing data, interactions, and sensitivity analyses) are in the appendix (pp 1–3). We then built a multivariable model including predictors that we considered to be clinically important for household tuberculosis, while also being easily recordable and consistent across diverse settings. We removed variables sequentially from the multivariable model that added little clinical or predictive value, assessed by examining regression coefficients and p values (threshold <0·2) for each variable in the context of the overall multivariable model. To derive a score, we used the coefficients for each variable included in the final model as weights.^11^ We calculated a score for each household using integers proportional to these weights and derived the predicted 3-year risk of household tuberculosis for each score value using the score regression coefficient and model constant. Because some of the variables included in our score might be unavailable in some settings, we also derived a simplified score including only very basic data that is likely to be available in all settings.

We evaluated the score's performance in both cohorts separately, including only households that had complete data. To characterise overall discrimination for predicting household tuberculosis, we calculated the concordance statistic (*c* statistic) with 95% CIs. Population quintiles of score were derived, and the risk of household tuberculosis in each quintile plotted with 95% CI. Time-to-tuberculosis curves were plotted and stratified by risk-score quintile.

We also evaluated the performance of the score to separately predict household tuberculosis diagnosed within the first 3 months after index patient treatment initiation (co-prevalent tuberculosis) and household tuberculosis diagnosed after the first 3 months (incident tuberculosis). We assessed calibration in the external validation cohort by comparing the mean predicted risk of household tuberculosis with the observed risk for each quintile. Because the score performed similarly in the derivation and external validation cohorts, we combined data from both cohorts to show how it could be used to inform clinical and public health decision making by calculating the score's sensitivity for all tuberculosis among contacts and specificity for any household tuberculosis, which were plotted against the predicted risk of household tuberculosis and the score distribution. We calculated the proportion of all tuberculosis cases among contacts captured by the highest scoring quarter, third, half, and two-thirds of households. We summarised these data for both the derivation and external validation cohorts separately.

### Role of the funding source

The funders had no role in study design, data collection, data analysis, data interpretation, or writing of the report. The corresponding author had full access to all the data in the study and had final responsibility for the decision to submit for publication.

## Results

**Figure 1:**
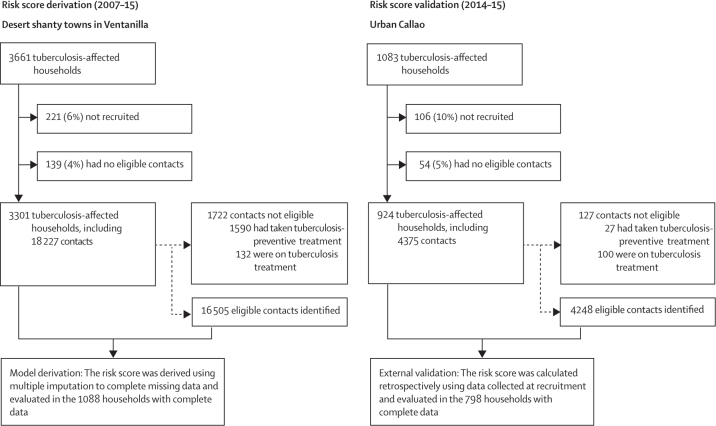
Study profile

Between Dec 12, 2007, and Dec 31, 2015, 3661 tuberculosis-affected households in Ventanilla and 1083 in Callao were recruited. 3301 (90%) of 3661 tuberculosis-affected households in Ventanilla were included in the derivation cohort, including 16 505 eligible contacts (figure 1). 924 (85%) of 1083 of tuberculosis-affected households in Callao were included in the external validation cohort, including 4248 eligible contacts. Differences in baseline characteristics between the two cohorts are shown in table 1.

**Table 1:** Characteristics of the derivation and external validation cohorts

		**Ventanilla derivation cohort**	**Callao validation cohort**	**p value**
Index patient characteristics*				
Age, years		··	··	0·055
	Median (IQR)	28 (20–42)	30 (21–44)	··
	Missing	4	1	··
Age, categories		··	··	0·085
	0–19 years	736 (22%)	179 (19%)	··
	20–49 years	1978 (60%)	560 (61%)	··
	≥50 years	583 (18%)	184 (20%)	··
Sex		··	··	0·47
	Male	2011 (61%)	575 (62%)	··
	Female	1290 (39%)	349 (38%)	··
	Missing	0	0	··
Type of tuberculosis and sputum smear grade		··	··	<0·0001
	Extra-pulmonary	469 (15%)	81 (9%)	··
	Pulmonary smear negative	796 (25%)	215 (24%)	··
	Pulmonary smear +	737 (23%)	253 (29%)	··
	Pulmonary smear ++	596 (18%)	169 (19%)	··
	Pulmonary smear +++	628 (19%)	160 (18%)	··
	Missing	75	46	··
Drug sensitivity		··	··	<0·0001
	Rifampicin sensitive	3012 (91%)	796 (86%)	··
	Rifampicin resistant	289 (9%)	127 (14%)	··
	Missing	0	0	··
Cough duration before diagnosis, days		··	··	0·027
	Median (IQR)	30 (7–60)	23 (9–45)	··
	Missing	103	51	··
Total symptom duration before diagnosis, days		··	··	0·028
	Median (IQR)	30 (12–60)	30 (15–60)	··
	Missing	96	45	··
Maximum number of hours any contact had spent with the index patient while they had any cough		··	··	0·0086
	Median (IQR)	120 (10–360)	150 (14–400)	··
	Missing	1064	5	··
Household characteristics*				
Access to piped water		··	··	<0·0001
	No	1206 (37%)	31 (3%)	··
	Yes	2071 (63%)	877 (97%)	··
	Missing	24	16	··
Access to a toilet in the household		··	··	<0·0001
	No	1480 (45%)	34 (4%)	··
	Yes	1797 (55%)	875 (96%)	··
	Missing	24	15	··
Wall material		··	··	<0·0001
	Adobe and other dirt	516 (16%)	56 (6%)	··
	Wood	1306 (40%)	116 (13%)	··
	Brick or cement	1459 (44%)	737 (81%)	··
	Missing	20	15	··
Floor material		··	··	<0·0001
	Dirt	966 (29%)	57 (6%)	··
	Cement or wood	2113 (64%)	621 (68%)	··
	Tiles or ceramic	202 (6%)	231 (25%)	··
	Missing	20	15	··
Total monthly income, PEN		··	··	<0·0001
	Median (IQR)	820 (560–1400)	1500 (1000–2400)	··
	Missing	70	17	··
Total monthly spending on food, PEN		··	··	<0·0001
	Median (IQR)	140 (105–175)	140 (120–210)	··
	Missing	38	15	··
Any household member a current drug user		··	··	0·19
	No	1093 (77%)	706 (80%)	··
	Yes	323 (23%)	182 (21%)	··
	Missing	1885	36	··
Any household member drinking alcohol to excess†		··	··	0·13
	No	963 (78%)	671 (75%)	··
	Yes	279 (22%)	227 (25%)	··
	Missing	2059	26	··
Level of schooling of female head of household‡		··	··	<0·0001
	Primary or no formal education	1011 (36%)	222 (26%)	··
	Secondary education incomplete	637 (23%)	184 (21%)	··
	Secondary education complete	952 (34%)	346 (40%)	··
	Higher education complete	191 (7%)	113 (13%)	··
	Missing	510	59	··
Household crowding§		··	··	<0·0001
	No	1446 (49%)	554 (61%)	··
	Yes	1511 (51%)	355 (39%)	··
	Missing	344	15	··
Contact characteristics (per household)*				
Number of contacts, median (IQR)		5 (3–7)	4 (3–6)	0·0013
Number of male contacts, median (IQR)¶		2 (1–3)	2 (1–3)	0·014
Number of children (aged under 15 years), median (IQR)||		1 (0–2)	1 (0–2)	0·0004
Number of people who previously had tuberculosis**		··	··	0·018
	0	2003 (61%)	576 (62%)	··
	1	878 (27%)	204 (21%)	··
	2	306 (9%)	104 (11%)	··
	3	79 (2%)	24 (3%)	··
	≥4	35 (1%)	16 (4%)	··
**Contact characteristics (individual)**††				
Age, years		··	··	<0·0001
	Median (IQR)	24 (12–40)	28 (14–46)	··
	Missing	279	39	··
Sex		··	··	0·91
	Male	8054 (49%)	2068 (49%)	··
	Female	8449 (51%)	2177 (51%)	··
	Missing	2	3	··
Weight		··	··	<0·0001
	Lower weight	904 (7%)	182 (4%)	··
	Normal weight	5568 (43%)	1782 (44%)	··
	Overweight	4577 (35%)	1489 (36%)	··
	Obese	1977 (15%)	638 (16%)	··
	Missing	3479	157	··

For a detailed description of variable definitions see the appendix (pp 1–3). Data are n or n (%) unless otherwise stated. Percentages were calculated using number of people with available data as the denominator. Statistical tests exclude participants with missing data. PEN=Peruvian Nuevos Soles.

* The Ventanilla derivation cohort consisted of 3301 households, and the Callao validation cohort consisted of 924 households.

† Household crowding was defined as an average of two or more people sleeping in each room (excluding bathrooms, kitchens, hallways, and any external buildings, such as garages).

‡ If there was no female head, the schooling level of the male head of the household was used.

§ Any member of the household drinking alcohol to excess was defined as the patient or any of their contacts reporting drinking alcohol to the extent that they were extremely drunk (eg, unable to remember events) at least once in the last month.

¶ Mean values were 2·44 (SD 2·33) in the derivation cohort and 2·23 (1·80) in the validation cohort.

|| Mean values were 1·45 (1·65) in the derivation cohort and 1·19 (SD 1·32) in the validation cohort.

** All contacts and previous household members, excluding the index patient.

†† The Ventanilla derivation cohort consisted of 16 505 individuals, and the Callao validation cohort consisted of 4248 individuals.

568 (3%) of 16 505 contacts from 430 (13%, 95% CI 12–14) of 3301 households included in the derivation cohort had tuberculosis within 3 years. The results of univariable analysis are shown in the appendix (p 5). In the final multivariable model, several index patient, household, and contact characteristics were independently associated with household tuberculosis (table 2). Index patient predictors were age, type of tuberculosis and sputum smear grade, and maximum number of hours a contact had spent with the index patient while they had any cough (table 2). Household predictors were drug use, schooling level of the female head of household, and spends less than the monthly median on food per person (table 2). Contact predictors were any of the contacts were children, number of adult contacts of lower weight, number of adult contacts of normal weight, and number of past or present household members (excluding the index patient) who previously had tuberculosis (table 2). The overall number of contacts, and the number of adult contacts who were overweight or obese, were not associated with household tuberculosis in multivariable analysis (data not shown). The index patient characteristic of cough duration, and the household-level characteristics of income per person, excessive alcohol consumption by any household member, and crowding, were all associated with household tuberculosis in univariable analysis but not in multivariable analysis (data not shown). Index patient resistance to rifampicin was not associated with household tuberculosis (OR 1·11, 95% CI 0·79–1·58).

**Table 2:** Multivariable logistic regression of predictors associated with household tuberculosis in the derivation cohort after multiple imputation

	**Adjusted odds ratio (95% CI)**	**p value**	**Regression coefficient**
**Index patient characteristics**			
Age of the index patient	1·47 (1·24–1·75)*	<0·0001	0·387
Type of tuberculosis and sputum smear grade	1·35 (1·24–1·47)*	<0·0001	0·299
Maximum number of hours any contact had spent with the index patient while they had any cough	1·39 (1·20–1·61)*	<0·0001	0·327
**Household characteristics**			
Level of schooling of female head of household†	1·15 (1·02–1·31)*	0·026	0·143
Spends less than the monthly median on food per person	1·38 (1·09–1·74)	0·0069	0·322
Any member of the household a current drug user	1·63 (1·19–2·24)	0·0027	0·488
**Contact characteristics**			
Any of the contacts children (aged <15 years)	1·57 (1·23–2·02)	0·0004	0·453
Number of lower-weight adult contacts (BMI <20·0 kg/m^2^)	1·55 (1·29–1·87)	<0·0001	0·440
Number of normal-weight adult contacts (BMI 20·0–24·9 kg/m^2^)	1·18 (1·10–1·27)	<0·0001	0·169
Number of past or present household members with previous tuberculosis (excluding the index patient)	1·27 (1·14–1·42)	<0·0001	0·240

**Figure 2:**
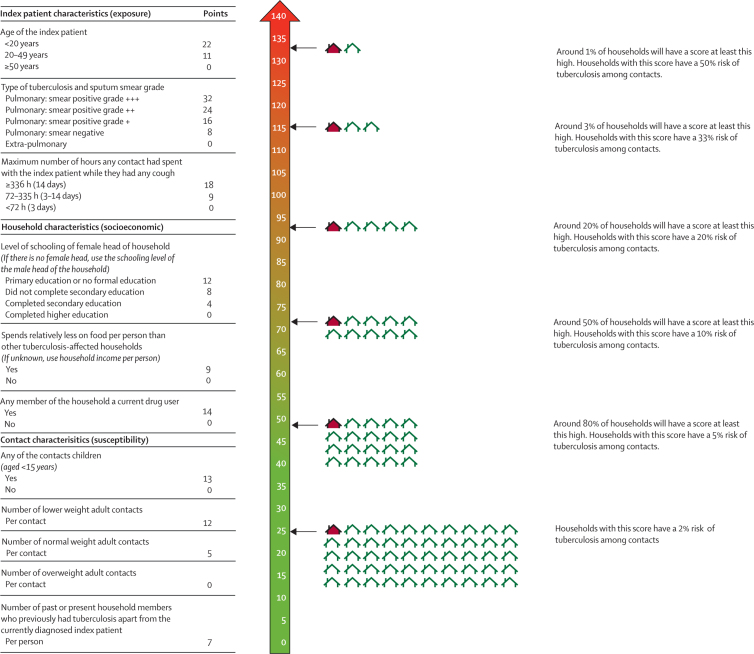
A risk score for field use

Full break down of each category and the points assigned in the risk score are shown in figure 2. Among the household characteristics, schooling level of the female head of household was associated with food spending (p_trend_<0·0001) but not drug use (p_trend_=0·26). Food spending was also associated with drug use (p<0·0001). For a detailed description of variables and analysis, including interactions investigated, see the appendix (pp 1–3). Odds ratios are adjusted for all other variables included in the multivariable model. BMI=body-mass index.

* Modelled as linear variables after examination as ordinal categorical variables in univariable regression. The odds ratio therefore indicates the increase in odds for each category of the variable.

† If there was no female head, the schooling level of the male head of household was used.

**Figure 3:**
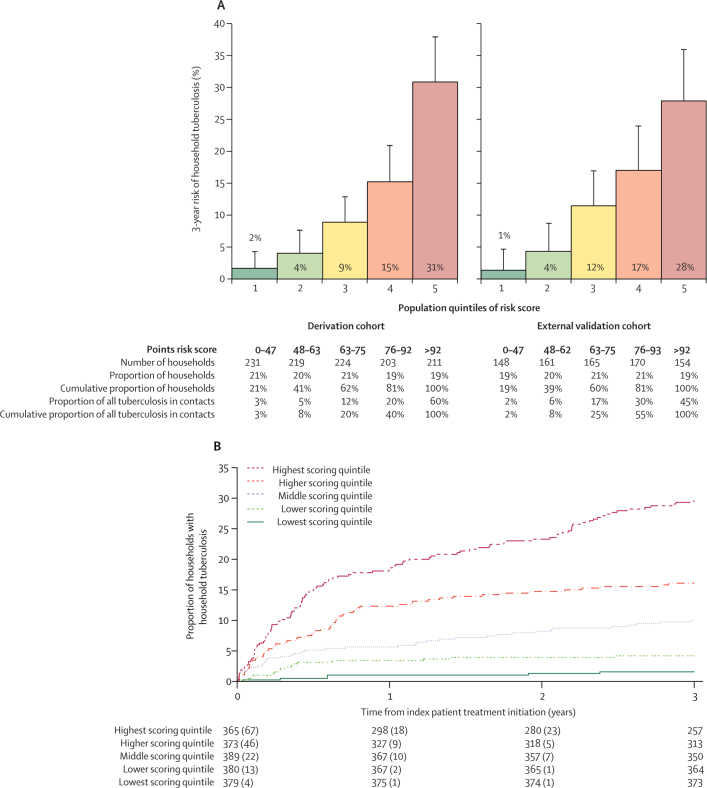
Risk of household tuberculosis and time to household tuberculosis in risk score quintiles (A) Tuberculosis risk score quintiles. Risk of household tuberculosis in population quintiles of risk score in both the derivation (n=1088) and external validation (n=798) cohorts. (B) Time-to-household tuberculosis stratified by risk score quintile. The curve was derived using data from both cohorts (n=1886). Separate curves for each cohort can be found in the appendix (p 6).

An example of how regression coefficients can be combined as integers into a score for field use is shown in figure 2. The proportion of missing data in the derivation cohort was small for the great majority of predictors included in the score (table 1). However, only 1416 (43%) of the 3301 households in the derivation cohort had data available on drug use, because it was not evaluated initially. Thus, 1088 (33%) of 3301 households had complete data for all predictors and were used to evaluate the score in the derivation cohort. The *c* statistic assessing the score's discrimination for household tuberculosis was 0·77 (95% CI 0·72–0·81). In the highest scoring quintile, the risk of household tuberculosis was 31% (95% CI 25–38; 65 of 211) compared with 9% (95% CI 6–13; 20 of 224) in the middle quintile and 2% (95% CI 0–4; four of 231) in the lowest scoring quintile (figure 3A).

In the validation cohort, 159 (4%) of 4248 contacts from 120 (13%, 95% CI 11–15) of 924 households had tuberculosis within 3 years. 798 (86%) of 924 households had complete data on all predictors (including drug use) and were used to evaluate the score. The *c* statistic was 0·75 (95% CI 0·70–0·79) and the risk of household tuberculosis was 28% (95% CI 21–36; 43 of 154) in the highest scoring quintile, 12% (7–17; 43 of 154) in the middle quintile, and 1% (0–5; two of 148) in the lowest scoring quintile (figure 3A). The score was well calibrated when comparing predicted versus observed risk in these quintiles (appendix p 6).

A time-to-tuberculosis curve for the whole cohort is shown in figure 3B and for the derivation and validation cohorts separately in the appendix (p 7). In both cohorts, the score predicted both co-prevalent and incident household tuberculosis (appendix p 4). The performance of the score did not change in a sensitivity analysis in which lower household food spending per person was replaced with lower household income per person: the *c* statistic was 0·76 (95% CI 0·72–0·81) for the derivation cohort and 0·75 (0·70–0·79) for the validation cohort (appendix p 3).

Considering households from both cohorts combined, 305 (4%) of 8545 contacts from 229 (12%) of 1886 households had tuberculosis. The sensitivity of the score for all tuberculosis cases among contacts and specificity for household tuberculosis are threshold specific and are shown in the appendix (p 8), with score distribution and predicted risk of household tuberculosis for each score. The highest scoring quarter of households (471 of 1886) captured 187 (61%) of 305 tuberculosis cases among contacts, a third of households (618 of 1886) captured 220 (72%) cases among contacts, half of households (945 of 1886) captured 259 (85%) cases, and two-thirds of households (1224 of 1886) captured 285 (93%) cases. These data are summarised for each cohort separately at different thresholds in the appendix (p 9).

**Figure 4:**
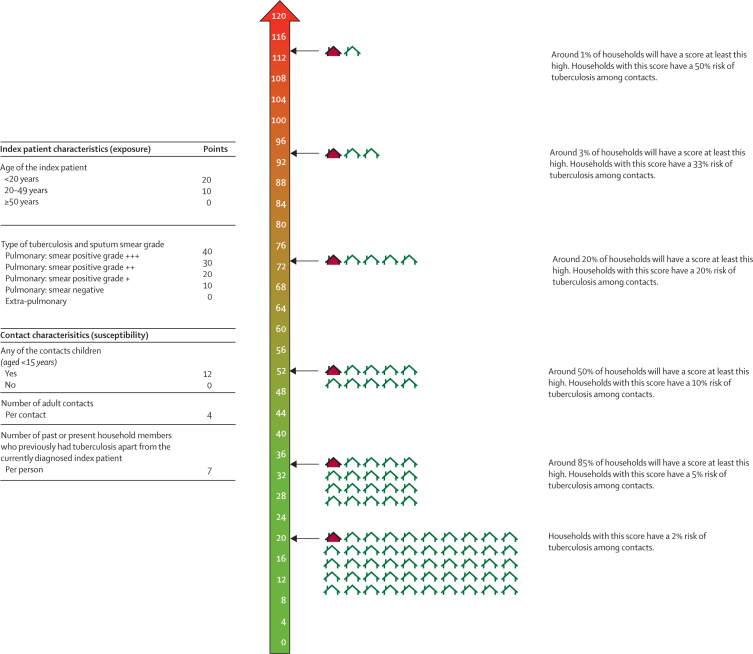
A simplified risk score for field use

Five variables were included in a multivariable model to derive a simplified risk score. Index patient predictors were age (adjusted OR 1·43, 95% CI 1·21–1·70) and type of tuberculosis and sputum smear grade (1·41, 1·29–1·53). Contact predictors were any of the contacts were children (1·55, 1·21–1·98), number of adult contacts (1·15, 1·10–1·20), and number of past or present household members (excluding the index patient) who previously had tuberculosis (1·28, 1·15–1·43; appendix p 10). An example of how the results of this multivariable model could be combined in a risk score is shown in figure 4. 3226 (98%) of 3301 households in the derivation cohort had complete data to evaluate the score and the *c* statistic was 0·71 (95% CI 0·68–0·73). 878 (95%) of 924 households in the validation cohort had complete data to evaluate the score and the *c* statistic was 0·74 (95% CI 0·70–0·79). Risk of tuberculosis and time-to-tuberculosis curves for population quintiles are shown in the appendix (p 11).

## Discussion

In this study of tuberculosis-affected households, which included more than 20 000 contacts from two independent cohorts, we derived and externally validated a household-level risk score that stratified households with large differences in the risk of tuberculosis occurring among contacts. This score combines data from readily collectable index patient, household, and contact characteristics into a model that could be used to target comprehensive biosocial, household-level interventions to households at highest risk of tuberculosis among contacts. Although these interventions should be considered for all tuberculosis-affected households, using a risk stratification approach could considerably improve their impact and cost-effectiveness, especially in resource-constrained settings.

We envisage that this score could be used at the time of index patient diagnosis to prioritise enhanced active case finding among contacts to detect tuberculosis earlier at a less infectious stage,^20^ preventive treatment, and social protection interventions to maximise access to health care and address poverty-related tuberculosis risk factors.^14^ Although these interventions have potential benefits for all tuberculosis-affected households, our score could be used by decision makers to prioritise interventions in several ways. For example, one approach in severely resource-constrained settings might be to focus on the highest scoring third of households, of which around 25% are likely to have household tuberculosis within 3 years. This is a very high proportion when considering that tuberculosis typically affects less than 1% of households in a community at a given time.^1^ In other settings, decision makers might use a more inclusive threshold. For example, prioritising the highest scoring two thirds of households would capture more than 90% of all tuberculosis among contacts. Prioritising a higher proportion of tuberculosis-affected households is likely to increase the epidemiological impact of household-level interventions and could be balanced against the availability of resources in specific settings.

Our strategy to derive a score was based on a preconceived framework of index patient, household, and contact factors that we considered to be potential predictors of household tuberculosis. We showed an approximately linear relationship between index patient type of tuberculosis and sputum smear grade and risk of household tuberculosis. In our previous work among adult contacts of patients with tuberculosis who were nearly all sputum smear positive,^11^ smear grade did not predict tuberculosis among individuals. However, in this larger study, a strength of which is the inclusion of patients with extra-pulmonary tuberculosis and bacteriologically unconfirmed pulmonary tuberculosis, type of tuberculosis and smear grade strongly predicted tuberculosis assessed at a household level. This finding might partly be because another strength of this study was the inclusion of children, whose principal exposure is more likely to be the current index patient. By contrast, our previous study only included adults, who might have had multiple exposures throughout their lives, reducing the importance of the infectiousness of the currently diagnosed index patient.^21^ Although an isolated sputum smear result is probably a crude measure of infectiousness, smear microscopy is still the most widely available diagnostic test globally, particularly in resource-constrained settings.^22^ Optimising microscopy by use of strategies such as viability staining^23^ to identify the most infectious patients could complement and further improve our score.

We showed in multivariable analysis that the maximum duration of exposure any contact had to the index patient while they had cough predicted household tuberculosis in a dose-dependent relationship, independently of index patient type of tuberculosis and sputum smear grade. We also showed an association between index patient age and risk of household tuberculosis. Households in which the index patient was younger than 20 years had the highest risk of tuberculosis, possibly because of undetected tuberculosis among adults in the household.^20^ Households in which the index patient was 50 years or older had the lowest risk of tuberculosis, possibly because these patients more commonly present with atypical symptoms,^24^ and because older people might be more commonly isolated when unwell. Although we did not observe an increased risk of household tuberculosis among households affected by rifampicin-resistant tuberculosis, the individual, household, and public health consequences of rifampicin-resistant tuberculosis strongly support the prioritisation of these households for interventions, independent of this risk score.

The other variables included in our score show that a biosocial approach is essential to ending the tuberculosis epidemic.^25^ Tuberculosis inequitably affects poorer households, principally in lower-income countries.^9^ Our finding that households in which the female head had less schooling, a general marker of household poverty,^26^ had a higher risk of household tuberculosis further supports this association between tuberculosis and poverty. Economic prosperity, leading to improved living conditions and better nutrition, is recognised as the most important driver of the reduction in tuberculosis incidence in western Europe during the pre-antibiotic era.^9^ Since then, multiple studies have shown the inverse association between tuberculosis incidence and socioeconomic development, including government spending on social protection.^27^, ^28^, ^29^ In this study, we extended our previous findings showing the role of nutritional factors in determining tuberculosis risk.^11^ Our results suggest that it is the nutritional status of contacts, and not the overall number of contacts, that best predicts which households will have a contact who has tuberculosis. For example, in multivariable analysis, the number of adult contacts of lower weight greatly increased risk of household tuberculosis, the number of adult contacts of normal weight somewhat increased risk, and the number of adult contacts who were overweight did not increase risk. Although we did not observe a clear linear relationship between the number of child contacts, or their weight, and household tuberculosis, households that included children were at substantially higher risk of household tuberculosis than those without children. This finding reinforces that optimal management of child contacts is needed to reduce childhood tuberculosis morbidity and mortality.^3^

Relatedly, we showed that households that spent relatively less on food per person had an increased risk of household tuberculosis. This variable might be an indicator of overall monetary poverty, reflected by the fact that the score performed equally well when replaced by income, and might also reflect food security and the quality of food consumed by household members. Furthermore, households that included anyone who used drugs were at higher risk of household tuberculosis, and households that included anyone who drank alcohol to excess were at higher risk of household tuberculosis in univariable analysis. Therefore, our score might be used to prioritise holistic interventions that aim to optimise the nutritional status of members of households at highest risk and address these harmful health behaviours. As well as reducing tuberculosis risk, improved nutrition and reduced harmful substance use are likely to have far reaching health benefits. We showed a dose-dependent increased risk of household tuberculosis among households previously affected by tuberculosis. This supports our approach of deriving a household-level risk score because tuberculosis clusters in households, frequently affecting multiple household members, which might be explained by an increased number of exposures for all contacts, previous tuberculosis conferring a high risk of subsequent tuberculosis among individuals,^30^ and by the fact that households previously affected by tuberculosis are likely to be poorer than households that have never been affected by tuberculosis.^31^

This study had some limitations. We might have underestimated the number of households in which a contact had tuberculosis because we did not actively follow-up contacts to establish tuberculosis diagnoses outside the study setting (eg, in private health facilities). However, our previous work^20^ showed that these cases account for a small proportion of the overall tuberculosis burden among contacts so are unlikely to have affected our results. We were unable to account for censoring of households that moved away or contacts who died. However, our experiences of working in this setting since 2002 suggest migration and death are rare.^20^ Similarly, we did not collect data on how variables changed over time because we aimed to derive a score that could be used at the time of index patient diagnosis using baseline data. Although a substantial proportion of households had missing data on some variables in the derivation cohort, we used robust multiple imputation methods to complete these data and facilitate increased power for score derivation, evaluated the score only among households with complete data, and externally validated the score in a distinct cohort of households for which the majority had complete data.

A strength of our approach is the inclusion of variables that are likely to be consistent and easily recordable by health workers across settings, which, given their role in determining tuberculosis risk, should be integrated into routinely collected data systems by national tuberculosis programmes. In settings where some of these data are not available, the simplified score including only five variables could be used with only a small reduction in performance. We did not have data on other risk factors, such as household ventilation or HIV infection among contacts. However, HIV prevalence in Peru is low (about 0·2% of women aged 15–49 years) and is unlikely to affect the interpretation of our results.^32^ In our previous study,^11^ we showed an increased tuberculosis risk among contacts exposed to indoor air pollution from cooking fuels. We were unable to investigate this variable for our current cohort because there has been a near universal shift to clean, gas cookers in our setting. The use of our score in other settings should, therefore, consider local epidemiology (including HIV prevalence), health behaviours, and household characteristics. Programmatic interventions targeted to tuberculosis-affected households aim to detect and prevent all cases of tuberculosis among contacts, irrespective of the source of infection. Therefore, we did not use molecular techniques to confirm transmission from index patients to contacts because it would not affect our findings.

In conclusion, we derived and externally validated a simple household-level risk score that stratifies tuberculosis-affected households with different risks of tuberculosis among contacts. The score had similar predictive performance in derivation and external validation cohorts, with excellent calibration in the external validation cohort, lending promise to the use, further validation, and impact evaluation of our score in other settings.
