# Supplementary material for: A household-level score to predict the risk of tuberculosis among contacts of patients with tuberculosis: a derivation and external validation prospective cohort study
Source: Lancet Infect Dis. 2020 Jan;20(1):110–22. doi: 10.1016/S1473-3099(19)30423-2 (PMC6928575; doi:10.1016/S1473-3099(19)30423-2)
Supplement: Supplementary appendix [file mmc1.pdf]

THE LANCET Infectious Diseases

Supplementary webappendix

This webappendix formed part of the original submission and has been peer reviewed. We post it as supplied by the authors.

Supplement to: Saunders MJ, Wingfield T, Datta S, et al. A household-level score to predict the risk of tuberculosis among contacts of patients with tuberculosis: a derivation and external validation prospective cohort study. *Lancet Infect Dis* 2019; published onlined Oct 30. https://doi.org/10.1016/S1473-3099(19)30423-2.

APPENDIX SUPPLEMENTARY METHODS

Contact investigation and preventive treatment

All contacts from tuberculosis-affected households included in this study were eligible for contact investigation provided free of direct charges at Peruvian Ministry of Health (MINSA)-run health posts. This principally entailed universal clinical assessment and sputum smear microscopy without culture testing of one or two sputum samples, collected irrespective of symptoms. Chest radiography was rarely done for contacts at the discretion of the assessing doctor. Contacts aged under 20 years were eligible to have a tuberculin skin test, although during the study this was rarely performed, partly because of a lack of tuberculin. Peruvian national guidelines focus tuberculosis preventive treatment on children aged under five years and other high-risk groups. In practice, coverage is low with only approximately 25% of eligible contacts initiating preventive treatment in a recent analysis.[1] This programmatic contact investigation and preventive treatment were not influenced by our study.

Variable definition, transformation and modelling strategy

We used logistic regression to investigate the association of index patient, household and contact characteristics with the outcome, household tuberculosis. Although we had data available on time to tuberculosis, we were not able to censor households that moved away or account for contacts that died. Furthermore, because we aimed to derive a score that could be used at the time of index patient diagnosis, we did not collect data on how variables changed over time. Therefore, we were not able to optimally use time to event analysis in this study. Although missing data were few for the majority of candidate predictor variables (Table 1), a number of potentially important variables had higher percentages of missing data because they were not collected initially from participants recruited to the derivation cohort. We therefore used multiple imputation with chained equations to replace missing values and facilitate modelling analysis including all households recruited to the derivation cohort.[2] The equations used to impute missing data included all potential predictor variables and the outcome variable (household tuberculosis, which was complete and therefore not imputed). We carried out ten imputations and used Rubin's rules to combine estimates across the imputed datasets.

Index patient characteristics. Index patient age was first examined in deciles, and, because we observed a higher risk of tuberculosis in households where the index patient was aged under 20 years, and a lower risk in households where the index patient was aged over 50 years, we created an ordinal categorical variable defining index patients aged over 50 years, 20-49 years, and under 20 years. Because the relationship between this three-tier categorical variable and log odds of household tuberculosis was approximately linear in univariable regression, we included this variable as a linear term in our multivariable model. We examined the association of index patient cough duration (of any type) and household tuberculosis as a continuous variable and created a dichotomous variable defining index patients as having had a longer cough if they had a cough for at least 21 days (three weeks). We chose this threshold because we felt it should be easily interpretable in a field setting and because after this point the risk of household tuberculosis stabilised. Index patient type of tuberculosis and sputum smear grade were analysed as an ordinal categorical variable.

Because the relationship between this variable and log odds of household tuberculosis was approximately linear in univariable regression, we included this variable as a linear term in our multivariable model. Index patient resistance to rifampicin was defined if the patient had microbiological evidence of resistance, or if they were prescribed a treatment regimen for rifampicin-resistant tuberculosis. Finally, we examined the association between the maximum number of hours any contact had spent with the index patient while they had cough (of any type) and household tuberculosis. Because this variable showed an approximately linear relationship with the outcome, we created a three-tier categorical variable choosing thresholds that we felt would be easily definable in a field setting.

Household characteristics. We calculated household monthly income and food spending per person sleeping in the household and dichotomised these variables by their median value to investigate their association with household tuberculosis. We chose this strategy in order to test our hypothesis that households with lower relative monetary indicators of poverty would have a higher risk of household tuberculosis, and because we felt that if these variables were to be included in a score, they would be most easily interpreted in other settings using this relative, dichotomised approach. We did not investigate the association of wall material, floor material, access to piped water and access to a toilet with household tuberculosis because we felt that these variables may not be applicable in other settings. Any member of the household being a drug user was defined if the patient or any of their contacts reported currently using any of: marihuana; cocaine (including an intermediary cocaine paste frequently smoked in Peru); ecstasy; heroin; or glue. Any member of the household drinking alcohol to excess was defined if the patient or any of their contacts reported drinking alcohol to the extent that they were extremely drunk (e.g. unable to remember events) at least once in the last month. The highest level of schooling attended by the female head of the household was examined as an ordinal categorical variable (higher, secondary completed, secondary incomplete and primary/no education) in univariable regression. If there was no female head of the household, data from the male head of the household were used. Because the relationship between this variable and log odds of household tuberculosis was approximately linear in univariable regression, we included this variable as a linear term in our multivariable model. Household crowding was defined if an average of two or more people were sleeping in each room (excluding bathrooms, kitchens, hallways and any external buildings such as garages).

Contact characteristics. Contacts were defined as children if aged under 15 years and adults if aged 15 years or older. If contacts were present at the time of index patient recruitment, they were weighed and measured. If they were not present, they were either telephoned to provide an estimate, or the index patient estimated the contact's weight and height based on local references. For some children who were not present, weight and height data were obtained from a health centre growth record book, if it was available. For adults aged 19 years or over, weight was defined as lower weight (BMI<20.0), normal weight (BMI=20.0-24.9), overweight (BMI=25.0-29.9), and obese (BMI≥30.0). For adults aged 15-18 years, BMI was adjusted using WHO reference standards as in our previous work and the same classification used.[3] For children aged 2-14 years, weight was defined using BMI-for-age Z scores derived from World Health Organization (WHO) reference standards as lower weight (Z score<-1), normal weight (Z score≥-1 and Z score<1), overweight (Z score≥1 and Z score<2) and obese (Z score≥2). For children aged under 2 years, weight was defined using WHO

weight-for-age Z scores using the same cut-offs as above. The number of contacts in a household who were lower weight, normal weight, overweight, and obese were calculated separately for adults and children and investigated initially as linear variables. Although we observed clear linear associations both for the number, and the weight, of adult contacts with household tuberculosis, weight of child contacts did not show a clear association with household tuberculosis. Furthermore, the risk of household tuberculosis increased if any of the contacts were children but did not increase linearly with the number of children living in the household. Therefore, because children are a priority group for tuberculosis prevention and care, we created a dichotomous variable if any of the contacts were children. Finally, because the relationship between the number of people who had previously had tuberculosis apart from the currently diagnosed index patient and household tuberculosis was approximately linear, we included this variable as a linear term in all our regression models. This variable included all contacts (including contacts who were not eligible because they were already taking treatment or received four weeks of isoniazid preventive treatment because of exposure to the current index patient) and previous household members who weren't currently living in the household.

Interactions

In our multivariable model we investigated three interaction terms: age of the index patient with type of tuberculosis and sputum smear grade; type of tuberculosis and sputum smear grade with maximum exposure any contact had; and low household food spending with number of lower weight adult contacts. These interactions terms did not improve the predictive performance of the model and were therefore not included in the final model.

Sensitivity analysis for the score

Because lower than the median monthly household food spending per person may be difficult to ascertain in some settings, we performed a sensitivity analysis replacing this variable in the risk score with lower than the median household monthly income per person. In this sensitivity analysis, having a household monthly income per person lower than median was given the same score weighting as having lower monthly household food spending per person.

1 Wingfield T, Tovar MA, Huff D, *et al.* A randomized controlled study of socioeconomic support to enhance tuberculosis prevention and treatment, Peru. *Bull World Health Organ* 2017; **95**: 270–80.

2 White IR, Royston P, Wood AM. Multiple imputation using chained equations: Issues and guidance for practice. *Stat Med* 2011; **30**: 377–99.

3 Saunders MJ, Wingfield T, Tovar MA, *et al.* A score to predict and stratify risk of tuberculosis in adult contacts of tuberculosis index cases: A prospective derivation and external validation cohort study. *Lancet Infect Dis* 2017; **17**: 1190–9.

APPENDIX SUPPLEMENTARY RESULTS

Co-prevalent tuberculosis

In the derivation cohort, 35% (150/430) of household tuberculosis was diagnosed in the first three months after the index patient initiated treatment. The score's C-statistic for this co-prevalent tuberculosis was 0.72 (95%CI=0.65-0.79). In the validation cohort, 33% (39/120) of household tuberculosis was diagnosed in the first three months after the index patient initiated treatment and the score's respective C-statistic was 0.73 (0.67-0.80).

Incident tuberculosis

In the derivation cohort, 65% (280/430) of household tuberculosis was diagnosed after the first three months after the index patient initiated treatment. The score's C-statistic for this incident tuberculosis was 0.78 (95%CI=0.73-0.83). In the validation cohort, 68% (81/120) of household tuberculosis was diagnosed after the first three months after the index patient initiated treatment and the score's respective C-statistic was 0.74 (0.69-0.80).

Table S1: Univariable logistic regression of factors associated with household tuberculosis in the derivation cohort (n=3,301)

		Unadjusted OR (95% CI)	p value
**Index patient characteristics**			
Age	50 years or over	Reference	Reference
	20-50 years	1.68 (1.22-2.32)	0.002
	Under 20 years	2.24 (1.57-3.18)	<0.0001
Sex	Female	Reference	Reference
	Male	0.96 (0.78-1.18)	0.7
Type of tuberculosis and sputum smear grade	Extra-pulmonary	Reference	Reference
	Pulmonary smear negative	1.94 (1.21-3.13)	0.006
	Pulmonary smear +	2.78 (1.75-4.44)	<0.0001
	Pulmonary smear ++	4.02 (2.54-6.38)	<0.0001
	Pulmonary smear +++	4.50 (2.85-7.10)	<0.0001
Drug sensitivity	Rifampicin sensitive	Reference	Reference
	Rifampicin resistant	1.11 (0.79-1.58)	0.5
Cough for greater than three weeks prior to diagnosis	No	Reference	Reference
	Yes	1.90 (1.51-2.38)	<0.0001
Maximum number of hours a contact had spent with the index patient while they had cough	<72 hours	Reference	Reference
	72 hours to 335 hours	1.55 (1.16-2.08)	0.004
	336 hours or more	2.30 (1.75-3.03)	<0.0001
**Household characteristics**			
Has relatively less income than other tuberculosis-affected households (lower than the median household monthly income)	No	Reference	Reference
	Yes	1.58 (1.27-1.96)	<0.0001
Spends relatively less on food per person than other tuberculosis-affected households (lower than the median household monthly spending on food per person)	No	Reference	Reference
	Yes	1.83 (1.47-2.28)	<0.0001
Any member of the household a current drug user	No	Reference	Reference
	Yes	2.03 (1.49-2.86)	<0.0001
Any member of the household drinking alcohol to excess	No	Reference	Reference
	Yes	1.38 (1.05-1.82)	0.02
Level of schooling of female head of household (if there was no female head, the schooling level of the male head of the household was used)	Higher complete	Reference	Reference
	Secondary incomplete	1.17 (0.66-2.07)	0.6
	Secondary complete	1.48 (0.84-2.67)	0.2
	Primary	1.77 (1.02-3.08)	0.04
Household crowding	No	Reference	Reference
	Yes	1.55 (1.25-1.91)	0.0001
**Contact characteristics (per household)**			
Number of contacts	Per contact	1.12 (1.09-1.16)	<0.0001
Any of the contacts children	No	Reference	Reference
	Yes	1.67 (1.32-2.11)	<0.0001
Number of lower weight adult contacts	Per contact	1.76 (1.48-2.01)	<0.0001
Number of normal weight adult contacts	Per contact	1.27 (1.19-1.35)	<0.0001
Number of overweight adult contacts	Per contact	1.17 (1.09-1.26)	<0.0001
Number of obese adult contacts	Per contact	1.05 (0.91-1.23)	0.5
Number of past or present household members who previously had tuberculosis apart from the currently diagnosed index patient (including all contacts and other previous household members)	Per person	1.44 (1.30-1.58)	<0.0001

For a detailed description of variables see above. OR indicates odds ratio. 95%CI indicates 95% confidence interval.

Figure S1: Calibration plot comparing observed three-year risk of household tuberculosis in score quintiles (plotted points) with average predicted three-year risk for the validation cohort (n=798). Error bars indicate 95% confidence intervals. The dotted line represents perfect prediction. The solid line is a linear trend line for the plotted points. The R^2 value for this trend line was 0.95.

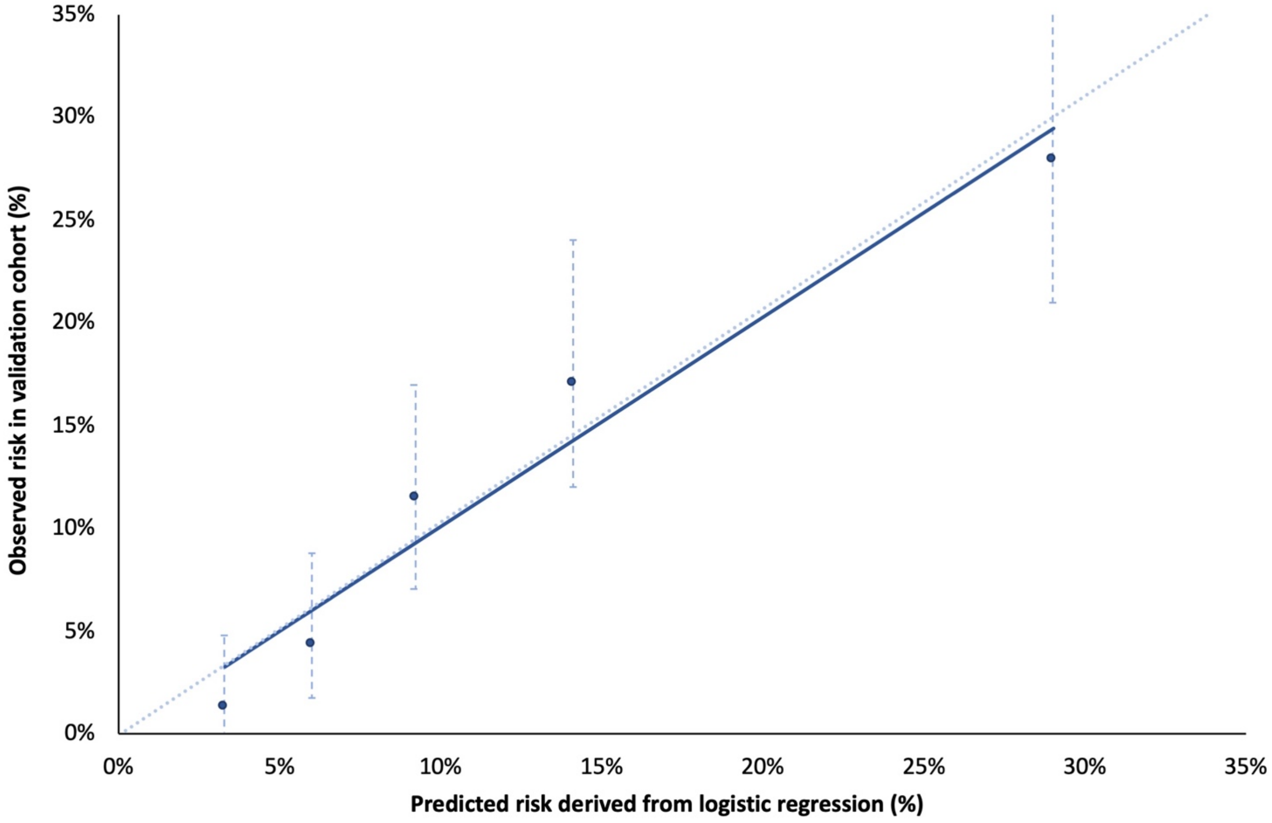

Figure S2: Time to tuberculosis curves for household tuberculosis stratified by risk score quintile

Figure S2a: Derivation cohort (n=1,088)

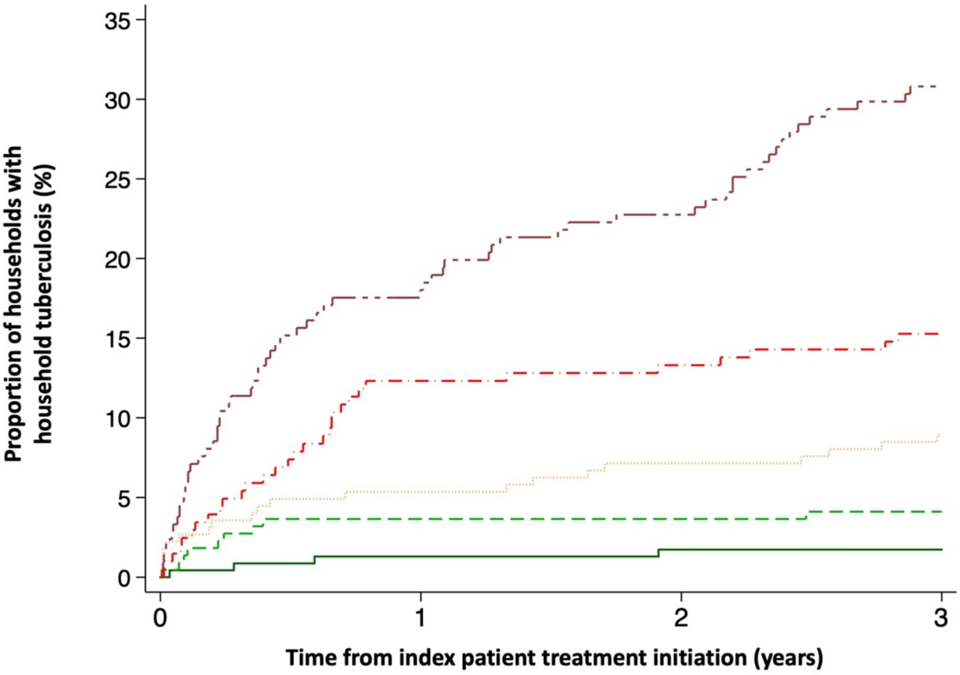

Highest scoring quintile	211 (38)	173 (10)	163 (17)	146
Higher scoring quintile	203 (25)	178 (2)	176 (4)	172
Middle scoring quintile	224 (12)	212 (4)	208 (4)	204
Lower scoring quintile	219 (8)	211 (0)	211 (1)	210
Lowest scoring quintile	231 (3)	228 (1)	227 (0)	227

Figure S2b: Validation cohort (n=798)

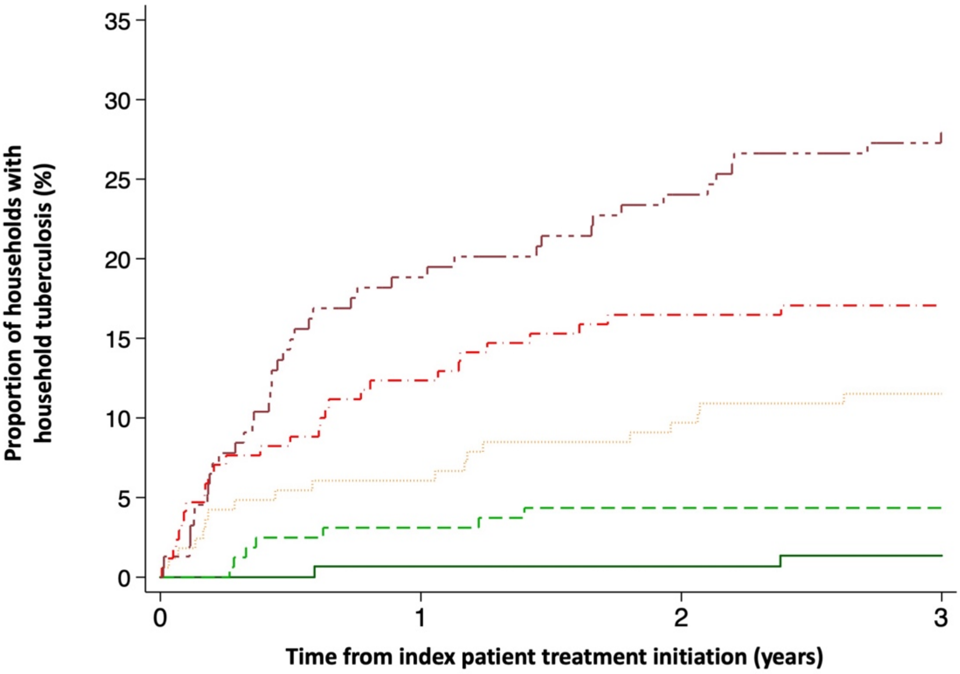

Highest scoring quintile	154 (29)	125 (8)	117 (6)	111
Higher scoring quintile	170 (21)	149 (7)	142 (1)	141
Middle scoring quintile	165 (10)	155 (6)	149 (3)	146
Lower scoring quintile	161 (5)	156 (2)	154 (0)	154
Lowest scoring quintile	148 (1)	147 (0)	147 (1)	146

Figure S3. Sensitivity for all tuberculosis among contacts, specificity for household tuberculosis, and predicted risk of household tuberculosis plotted against the population distribution of the risk score including households from both cohorts (n=1,886).

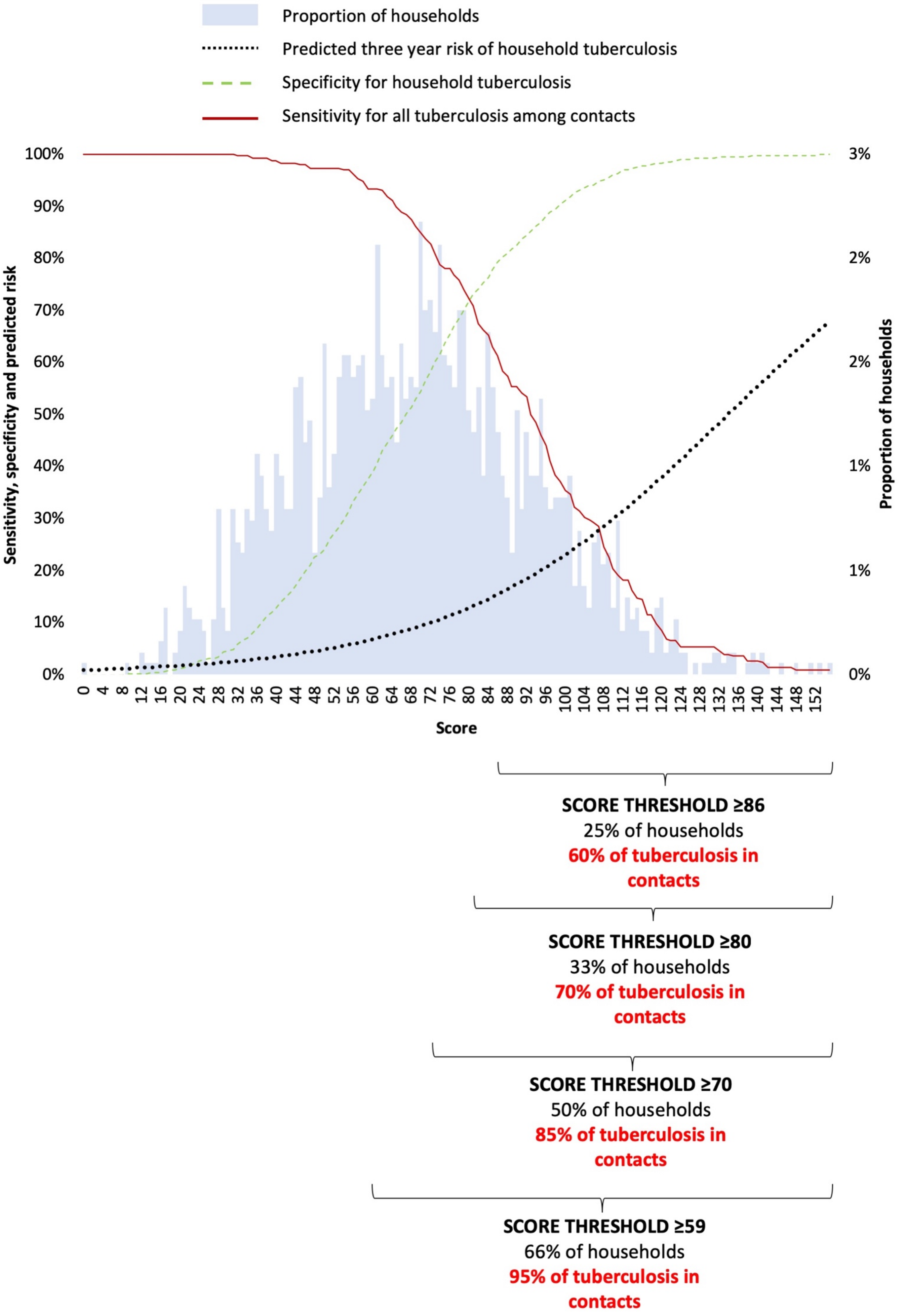

Table S2: Implications for decision making at different score thresholds stratified by cohort

	Derivation cohort	External validation cohort	Both cohorts together
**Overall performance**			
Number of households with complete data available for score	1,088	798	1,886
Total number of contacts in these households	4,860	3,685	8,545
Proportion of contacts who had tuberculosis within three years	3.5% (172/4,860)	3.6% (133/3,685)	3.6% (305/8,545)
Absolute risk of household tuberculosis	12% (129/1,088)	13% (100/798)	12% (229/1,886)
Overall C-statistic for predicting household tuberculosis (95% confidence interval)	0.77 (0.72-0.81)	0.75 (0.70-0.79)	0.76 (0.73-0.79)
**Score threshold 59**			
Proportion of households with a score at least as high	65% (703/1,088)	68% (541/798)	66% (1,224/1,886)
Proportion of total number of contacts in these households	74% (3,596/4,860)	77% (2,835/3,685)	75% (6,431/8,545)
Proportion of all tuberculosis in contacts within three years	93% (160/172)	94% (125/133)	93% (285/305)
Absolute risk of household tuberculosis above this threshold	17% (118/703)	17% (94/541)	17% (212/1,224)
**Score threshold 70**			
Proportion of households with a score at least as high	49% (532/1,088)	52% (413/798)	50% (945/1,886)
Proportion of total number of contacts in these households	60% (2,906/4,860)	63% (2,304/3,685)	61% (5,210/8,545)
Proportion of all tuberculosis in contacts within three years	84% (145/172)	86% (114/133)	85% (259/305)
Absolute risk of household tuberculosis above this threshold	19% (103/532)	21% (85/413)	20% (188/945)
**Score threshold 80**			
Proportion of households with a score at least as high	32% (350/1,088)	34% (268/798)	33% (618/1,886)
Proportion of total number of contacts in these households	43% (2,068/4,860)	46% (1,709/3,685)	44% (3,777/8,545)
Proportion of all tuberculosis in contacts within three years	75% (129/172)	68% (91/133)	72% (220/305)
Absolute risk of household tuberculosis above this threshold	25% (87/360)	24% (65/268)	25% (152/618)
**Score threshold 86**			
Proportion of households with a score at least as high	25% (269/1,088)	25% (202/798)	25% (471/1,886)
Proportion of total number of contacts in these households	34% (1,650/4,860)	38% (1,386/3,685)	36% (3,306/8,545)
Proportion of all tuberculosis in contacts within three years	66% (114/172)	55% (73/133)	61% (187/305)
Absolute risk of household tuberculosis above this threshold	28% (74/269)	26% (53/202)	27% (127/471)
**Score threshold 96**			
Proportion of households with a score at least as high	15% (163/1,088)	15% (119/798)	15% (282/1,886)
Proportion of total number of contacts in these households	22% (1,089/4,860)	24% (884/3,685)	23% (1,976/8,545)
Proportion of all tuberculosis in contacts within three years	51% (88/172)	35% (46/133)	44% (134/305)
Absolute risk of household tuberculosis above this threshold	33% (53/163)	27% (32/119)	30% (85/282)

Table S3: Multivariable logistic regression of predictors associated with household tuberculosis in the derivation cohort after multiple imputation (n=3,301) – simplified model

		Adjusted OR (95% CI)	p value	Regression coefficient	Points assigned in risk score[a]
**Index patient characteristics**					
Age of the index patient	Over 50 years	Reference	Reference	Reference	0
	20-49 years	1.43 (1.21-1.70)*	<0.0001	0.361	10
	Under 20 years				20
Type of tuberculosis and sputum smear grade	Extra-pulmonary	Reference	Reference	Reference	0
	Pulmonary smear negative	1.41 (1.29-1.53)*	<0.0001	0.340	10
	Pulmonary smear +				20
	Pulmonary smear ++				30
	Pulmonary smear +++				40
**Contact characteristics**					
Any of the contacts children (aged under 15 years)	No	Reference	Reference	Reference	0
	Yes	1.55 (1.21-1.98)	0.0004	0.438	12
Number of adult contacts	Per additional contact	1.15 (1.10-1.20)	<0.0001	0.143	4
Number of people who previously had tuberculosis apart from the currently diagnosed index patient (all contacts and other previous household members)	Per additional person	1.28 (1.15-1.43)	<0.0001	0.246	7

OR indicates odds ratio. 95%CI indicates 95% confidence interval. BMI indicates body mass index.
[a]To calculate the number of points to be included in the score, regression coefficients were multiplied by a constant (6.99) and then multiplied by four and rounded to the nearest whole number.
*Age; and type of tuberculosis and sputum smear grade were modelled as linear variables after examination as ordinal categorical variables in univariable regression. The OR therefore indicates the increase in odds for each level of the variable. For a detailed description of variables and analysis, including interactions investigated, see the appendix, pages 1-3.

Figure S4a: Risk of household tuberculosis in population quintiles of the simplified risk score in both the derivation (n=3,226) and external validation cohorts (n=878)

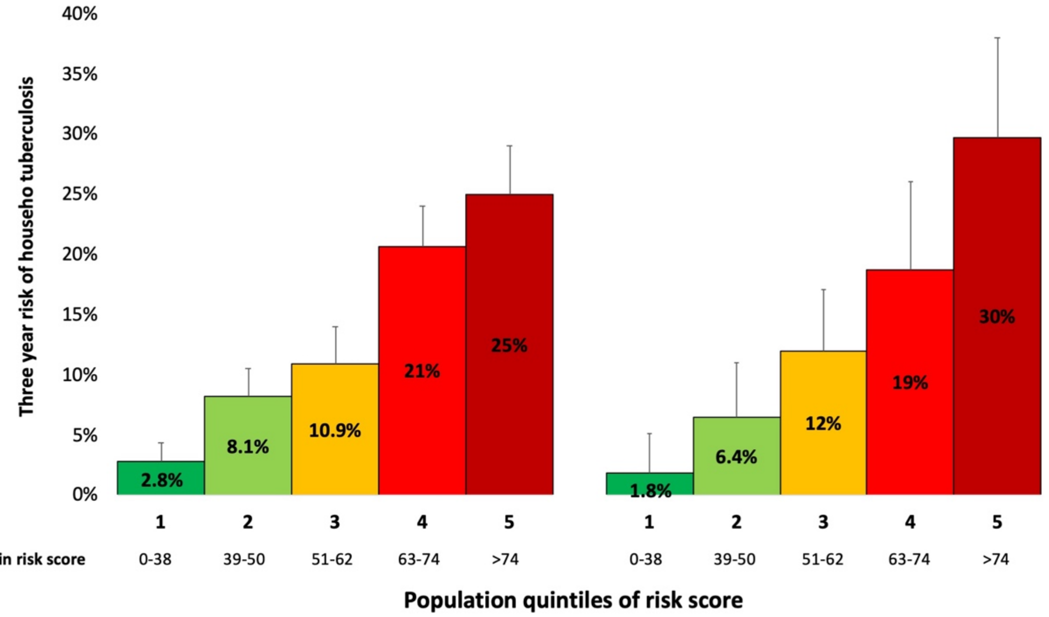

	Derivation cohort					External validation cohort				
Number of households	689	610	660	639	598	170	187	218	155	148
Proportion of households	21%	20%	20%	20%	19%	19%	21%	25%	18%	17%
Cumulative proportion of households	21%	40%	61%	81%	100%	19%	40%	65%	83%	100%
Proportion of all tuberculosis in contacts	3.4%	10%	14%	30%	42%	2.9%	8.6%	20%	26%	43%
Cumulative proportion of all tuberculosis in contacts	3.4%	14%	28%	58%	100%	2.9%	11%	31%	57%	100%

Figure S4b: Time to household tuberculosis stratified by simplified risk score quintile including data from both cohorts (n=4,104)

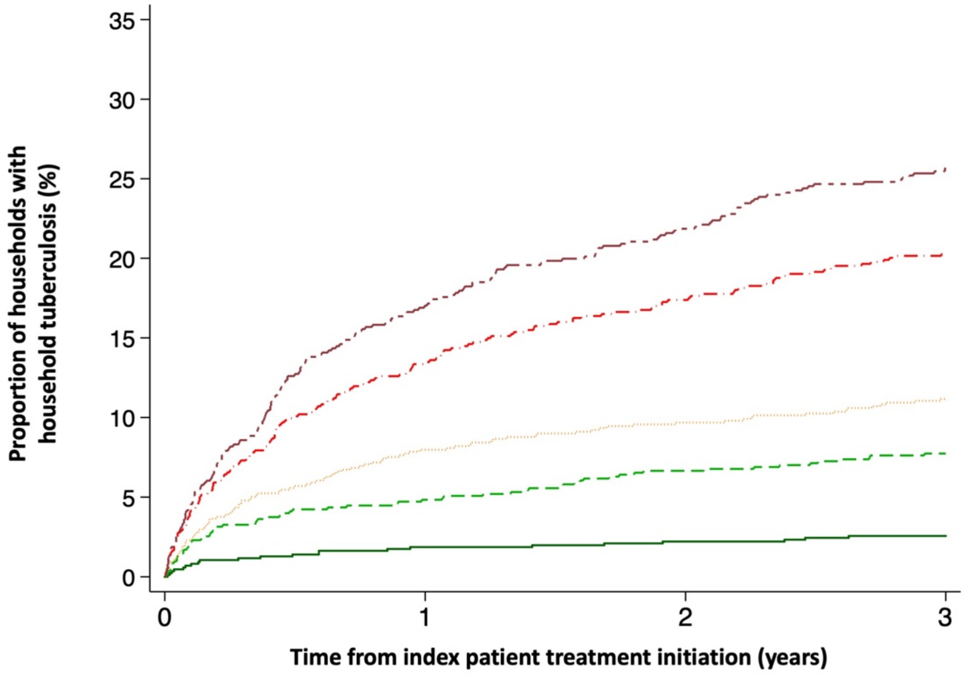

Highest scoring quintile	746 (127)	619 (36)	583 (30)	554
Higher scoring quintile	794 (106)	688 (32)	656 (23)	633
Middle scoring quintile	878 (70)	808 (15)	793 (13)	780
Lower scoring quintile	827 (40)	787 (15)	772 (9)	763
Lowest scoring quintile	859 (16)	843 (3)	840 (3)	837
